# Crystal structure of 4-meth­oxy-*N*-[(pyrrolidin-1-yl)carbo­thio­yl]benzamide

**DOI:** 10.1107/S2056989015003813

**Published:** 2015-03-04

**Authors:** Khairi Suhud, Lee Yook Heng, Siti Aishah Hasbullah, Musa Ahmad, Mohammad B. Kassim

**Affiliations:** a School of Chemical Sciences and Food Technology, Faculty of Science and Technology, Universiti Kebangsaan Malaysia, 43600 Selangor, Malaysia; b Department of Chemistry, Mathematics & Natural Science Faculty, Universitas Syiah Kuala, Banda Aceh, 23111, Indonesia; c Chemical Technology Program, Faculty of Science Technology, Universiti Sains Islam Malaysia, Bandar Baru Nilai, 71800 Nilai, Negeri Sembilan, Malaysia; d Fuel Cell Institute, Universiti Kebangsaan Malaysia, 43600 Selangor, Malaysia

**Keywords:** crystal structure, benzoyl­thio­urea, pyrrolidine, thio­urea, benzamide, hydrogen bonding

## Abstract

In the title compound, C_13_H_16_N_2_O_2_S, the pyrrolidine ring has a twisted conformation on the central –CH_2_–CH_2_– bond. Its mean plane is inclined to the 4-meth­oxy­benzoyl ring by 72.79 (15)°. In the crystal, mol­ecules are linked by N—H⋯O and C—H⋯O hydrogen bonds to the same O-atom acceptor, forming chains along [001]. The chains are linked *via* slipped parallel π–π inter­actions [inter-centroid distance = 3.7578 (13) Å], forming undulating slabs parallel to (100).

## Related literature   

For thio­urea derivatives containing a carbono­thioyl *R*–C(=O)—N(H)—C(=S)—N functional group, where *R* is an alkyl or aryl group, see: Arslan *et al.* (2006 ▸). For copper(II) complexes of similar compounds, see: Kulcu *et al.* (2005 ▸); Tan *et al.* (2014 ▸). For the biological properties of coordination complexes of such compounds, see: Rodríguez-Fernandez *et al.* (2005 ▸); Cikla *et al.* (2010 ▸). For the crystal structures of similar compounds, see: Al-abbasi *et al.* (2011 ▸, 2012 ▸); Md Nasir *et al.* (2011 ▸); Hassan *et al.* (2008 ▸, 2009 ▸).
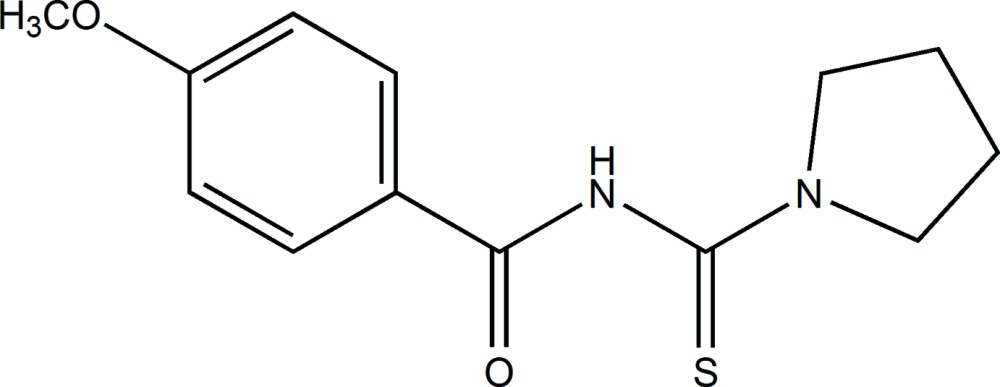


## Experimental   

### Crystal data  

C_13_H_16_N_2_O_2_S
*M* *_r_* = 264.34
Monoclinic, 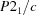

*a* = 11.8548 (12) Å
*b* = 11.4463 (11) Å
*c* = 9.8317 (9) Å
β = 93.124 (3)°
*V* = 1332.1 (2) Å^3^
*Z* = 4
Mo *K*α radiation
μ = 0.24 mm^−1^
*T* = 296 K
0.50 × 0.41 × 0.15 mm

### Data collection  

Bruker SMART APEX CCD area-detector diffractometer
Absorption correction: multi-scan (*SADABS*; Bruker, 2007 ▸) *T*_min_ = 0.890, *T*_max_ = 0.965
17732 measured reflections
2763 independent reflections
2140 reflections with *I* > 2σ(*I*)
*R*_int_ = 0.043

### Refinement  

*R*[*F* ^2^ > 2σ(*F* ^2^)] = 0.049
*wR*(*F* ^2^) = 0.130
*S* = 1.07
2763 reflections
169 parameters
H atoms treated by a mixture of independent and constrained refinement
Δρ_max_ = 0.26 e Å^−3^
Δρ_min_ = −0.23 e Å^−3^

### 

Data collection: *SMART* (Bruker, 2007 ▸); cell refinement: *SAINT* (Bruker, 2007 ▸); data reduction: *SAINT*; program(s) used to solve structure: *SHELXS97* (Sheldrick, 2008 ▸); program(s) used to refine structure: *SHELXL97* (Sheldrick, 2008 ▸); molecular graphics: *SHELXTL* (Sheldrick, 2008 ▸); software used to prepare material for publication: *SHELXTL*, *PLATON* (Spek, 2009 ▸) and *publCIF* (Westrip, 2010 ▸).

## Supplementary Material

Crystal structure: contains datablock(s) I. DOI: 10.1107/S2056989015003813/su5083sup1.cif

Structure factors: contains datablock(s) I. DOI: 10.1107/S2056989015003813/su5083Isup2.hkl

Supporting information file. DOI: 10.1107/S2056989015003813/su5083Isup3.cml

**Fig. 1.**
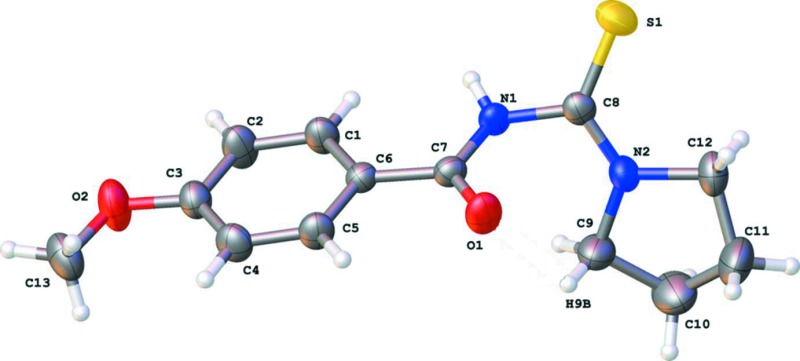
A view of the molecular structure of the title compound, with atom labelling. Displacement ellipsoids are drawn at the 50% probability level.

**Fig. 2.**
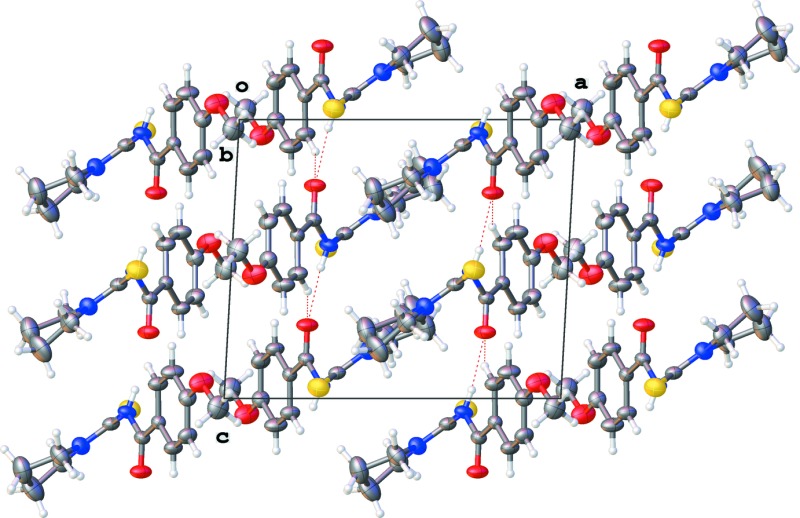
A view along the x axis of the crystal packing of the title compound. Hydrogen bonds are shown as dashed lines (see Table 1 for details).

. DOI: 10.1107/S2056989015003813/su5083fig1.tif
A view of the mol­ecular structure of the title compound, with atom labelling. Displacement ellipsoids are drawn at the 50% probability level.

x . DOI: 10.1107/S2056989015003813/su5083fig2.tif
A view along the *x* axis of the crystal packing of the title compound. Hydrogen bonds are shown as dashed lines (see Table 1 for details).

CCDC reference: 1051012

Additional supporting information:  crystallographic information; 3D view; checkCIF report

## Figures and Tables

**Table 1:** Hydrogen-bond geometry (, )

*D*H*A*	*D*H	H*A*	*D* *A*	*D*H*A*
N1H1*A*O1^i^	0.83(3)	2.11(3)	2.927(2)	170(2)
C1H1O1^i^	0.93	2.50	3.350(3)	152

Symmetry code: (i) 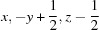.

## supplementary crystallographic information

### S1. Synthesis and crystallization

Benzoyl chloride (0.01 mol) was slowly added to ammonium thio­cyanate (0.01 mol) in acetone and the mixture was stirred for 30 min at room temperature. A white precipitate of ammonium chloride was filtered off. The filtrate was cooled in an ice bath (278-283 K) for about 15 min. Then, a cold solution (5-10°C) of pyrrolidine (0.01 mol) in acetone was added to the benzoyl iso­thio­cyanate and the mixture was left for 3 h at room temperature. A yellowish solution was formed and the mixture was filtered into a beaker containing some ice cubes. The yellow residue was washed with cold water followed pale-yellow block-like crystals (yield: 85%; m.p. 397-399 K). IR(KBr, cm^-1^) ν(-NH) 3389; (O—CH_3_) = 2967; ν(C═O_aliphatic_) = 1716, ν(C—C_benzene_) = 1651 and 1424; ν(C═O_stretching_) = 1311 and ν(C═S) = 1210 cm^-1^.

### S2. Refinement

Crystal data, data collection and structure refinement details are summarized in Table 3. The NH H atom, H1A, was located in a difference Fourier map and freely refined. The C-bound H atoms were included in calculated positions and treated as riding atoms: C–H = 0.93 – 0.97 Å with U_iso_(H) = 1.5U_eq_(C) for methyl H atoms and = 1.2U_eq_(C) for other H atoms.

### S3. Comment

The title compound, is a derivative of a thiourea compound containing a carbonothioyl *R*–C(═O)—N(H)—C(═S)—N functional group (Arslan *et al.*, 2006), where R can be either alkyl or aryl group. The C═O and C═S groups can serve as coordination sites upon reaction with metal ions. Such complexes have been found to be biologically active, for example anti-bacterial, anti-fungal (Rodríguez-Fernandez *et al.*, 2005), and anti-cancer (Cikla *et al.*, 2010). The title compound is similar to the previously reported derivatives namely 1,1-diethyl-3-(4-methoxybenzoyl)thiourea (Al-abbasi *et al.*, 2011) and 3-(3-methoxybenzoyl)-1,1-diphenylthiourea (Md Nasir *et al.*, 2011).

Reaction of these organic compounds *cis*-bis[4-chloro-N-(pyrrolidine-1-carbothioyl)-benzamido] with Cu(II) formed a stable complex compound with a 1:2 ratio. The complex was tested for anti-bacterial activity (Kulcu *et al.*, 2005). Similarly, a 1:3 ratio between copper(II)acetate and 1-benzoyl-(3,3-disubstituted)thiourea derivatives gave a series of copper(III) complexes in which the 1-benzoyl-(3,3-disubstituted)thiourea derivatives were deprotonated prior to the complexation reaction. Hence, 1-benzoyl-(3,3-disubstituted)thiourea derivatives behaved as a bidentate chelate through (O,S) coordination to give neutral cobalt(III) complexes (Tan *et al.*, 2014).Herein we report on the crystal structure of the title compound (MPCB), and compare it to previously reported carbonyl thioureas.

In the title compound, Fig. 1, the 4-methoxybenzoyl and pyrrolidine fragments adopting a *trans-cis* conformation with respect to the thiono S atom across the C8—N1 bond. The pyrolidine ring (N2/C2-C9) has a twisted conformation on the C10-C11 bond. The benzamide fragment (C1-C7/O1/N1) is approximately planar with a maximum deviation for atom O1 [0.045 (2)°], and it is twisted with respect to the thiourea fragment (S1/N1/N2/C8/C12) [maximum deviation of -0.034 (2)° for N2] with a dihedral angle of 61.81 (7)°.

The C═O [1.220 (2) Å] and C=S [1.662 (2) Å] bond lengths are comparable to those reported for propyl 2-(3-benzoylthioureido)acetate [1.220 (3) Å, 1.658 (3) Å, respectively] (Hassan *et al.*, 2008) and methyl 2-(3-benzoylthioureido)acetate [1.223 (5) Å, 1.661 (4) Å, respectively] (Hassan *et al.*, 2009). Other bond lengths and angles in the molecule are comparable those reported for N-(pyrrolidin-1-ylcarbothioyl) benzamide (Al-abbasi *et al.*, 2012).

In the crystal, molecules are linked by bifurcated N-H···O and C-H···O hydrogen bond forming chains along the c-axis direction (Table 1 and Fig. 2). Further stabilization is afforded by slipped parallel π-π stacking interactions involving the (C1-C6) benzene ring [Cg1···Cg1^i^ = 3.7578 (13) Å; inter-planar distance = 3.6228 (9) Å; slippage = 0.998 Å; symmetry code: (i) -x,+2, -y+1, -z], forming undulating slabs parallel to (100).

### Crystal data

**Table:**

C_13_H_16_N_2_O_2_S	*F*(000) = 560
*M**_r_* = 264.34	*D*_x_ = 1.318 Mg m^−^^3^
Monoclinic, *P*2_1_/*c*	Melting point = 397–399 K
Hall symbol: -P 2ybc	Mo *K*α radiation, λ = 0.71073 Å
*a* = 11.8548 (12) Å	θ = 3.2–26.5°
*b* = 11.4463 (11) Å	µ = 0.24 mm^−^^1^
*c* = 9.8317 (9) Å	*T* = 296 K
β = 93.124 (3)°	Block, pale-yellow
*V* = 1332.1 (2) Å^3^	0.50 × 0.41 × 0.15 mm
*Z* = 4	

### Data collection

**Table:**

Bruker SMART APEX CCD area-detector diffractometer	2763 independent reflections
Radiation source: fine-focus sealed tube	2140 reflections with *I* > 2σ(*I*)
Graphite monochromator	*R*_int_ = 0.043
ω scan	θ_max_ = 26.5°, θ_min_ = 3.2°
Absorption correction: multi-scan (*SADABS*; Bruker, 2007)	*h* = −14→14
*T*_min_ = 0.890, *T*_max_ = 0.965	*k* = −14→14
17732 measured reflections	*l* = −12→12

### Refinement

**Table:**

Refinement on *F*^2^	Secondary atom site location: difference Fourier map
Least-squares matrix: full	Hydrogen site location: inferred from neighbouring sites
*R*[*F*^2^ > 2σ(*F*^2^)] = 0.049	H atoms treated by a mixture of independent and constrained refinement
*wR*(*F*^2^) = 0.130	*w* = 1/[σ^2^(*F*_o_^2^) + (0.0485*P*)^2^ + 1.055*P*] where *P* = (*F*_o_^2^ + 2*F*_c_^2^)/3
*S* = 1.07	(Δ/σ)_max_ < 0.001
2763 reflections	Δρ_max_ = 0.26 e Å^−^^3^
169 parameters	Δρ_min_ = −0.23 e Å^−^^3^
0 restraints	Extinction correction: *SHELXL97* (Sheldrick, 2008), Fc^*^=kFc[1+0.001xFc^2^λ^3^/sin(2θ)]^-1/4^
Primary atom site location: structure-invariant direct methods	Extinction coefficient: 0.031 (3)

### Special details

**Table:**

Geometry. All e.s.d.'s (except the e.s.d. in the dihedral angle between two l.s. planes) are estimated using the full covariance matrix. The cell e.s.d.'s are taken into account individually in the estimation of e.s.d.'s in distances, angles and torsion angles; correlations between e.s.d.'s in cell parameters are only used when they are defined by crystal symmetry. An approximate (isotropic) treatment of cell e.s.d.'s is used for estimating e.s.d.'s involving l.s. planes.
Refinement. Refinement of *F*^2^ against ALL reflections. The weighted *R*-factor *wR* and goodness of fit *S* are based on *F*^2^, conventional *R*-factors *R* are based on *F*, with *F* set to zero for negative *F*^2^. The threshold expression of *F*^2^ > σ(*F*^2^) is used only for calculating *R*-factors(gt) *etc*. and is not relevant to the choice of reflections for refinement. *R*-factors based on *F*^2^ are statistically about twice as large as those based on *F*, and *R*- factors based on ALL data will be even larger.

### Fractional atomic coordinates and isotropic or equivalent isotropic displacement parameters (Å^2^)

**Table:**

	*x*	*y*	*z*	*U*_iso_*/*U*_eq_	
S1	0.72135 (6)	0.00137 (5)	0.03914 (6)	0.0491 (2)	
O1	0.76464 (14)	0.29334 (14)	0.26290 (14)	0.0456 (4)	
O2	0.93009 (16)	0.73574 (15)	−0.05668 (17)	0.0582 (5)	
N1	0.71420 (15)	0.23446 (15)	0.04921 (17)	0.0345 (4)	
N2	0.58223 (15)	0.13540 (14)	0.17024 (18)	0.0383 (4)	
C1	0.80147 (19)	0.45074 (19)	−0.0520 (2)	0.0386 (5)	
H1	0.7689	0.3985	−0.1151	0.046*	
C2	0.8420 (2)	0.5562 (2)	−0.0950 (2)	0.0472 (6)	
H2	0.8365	0.5748	−0.1872	0.057*	
C3	0.89105 (18)	0.63501 (18)	−0.0028 (2)	0.0387 (5)	
C4	0.89724 (19)	0.60796 (19)	0.1342 (2)	0.0421 (5)	
H4	0.9283	0.6610	0.1973	0.051*	
C5	0.85703 (19)	0.50171 (18)	0.1772 (2)	0.0383 (5)	
H5	0.8623	0.4834	0.2695	0.046*	
C6	0.80889 (15)	0.42189 (16)	0.08530 (18)	0.0290 (4)	
C7	0.76284 (16)	0.31244 (17)	0.14072 (18)	0.0310 (4)	
C8	0.66841 (17)	0.12705 (17)	0.09137 (18)	0.0320 (4)	
C9	0.5215 (2)	0.2428 (2)	0.2040 (3)	0.0504 (6)	
H9A	0.5074	0.2912	0.1240	0.060*	
H9B	0.5640	0.2877	0.2731	0.060*	
C10	0.4126 (2)	0.1985 (3)	0.2569 (4)	0.0705 (8)	
H10A	0.3559	0.1871	0.1832	0.085*	
H10B	0.3835	0.2521	0.3229	0.085*	
C11	0.4468 (3)	0.0836 (3)	0.3227 (4)	0.0747 (9)	
H11A	0.4820	0.0959	0.4129	0.090*	
H11B	0.3819	0.0329	0.3299	0.090*	
C12	0.5294 (2)	0.0320 (2)	0.2289 (3)	0.0469 (6)	
H12A	0.5851	−0.0159	0.2787	0.056*	
H12B	0.4910	−0.0150	0.1585	0.056*	
C13	0.9838 (2)	0.8187 (2)	0.0323 (3)	0.0582 (7)	
H13A	0.9295	0.8506	0.0911	0.087*	
H13B	1.0149	0.8804	−0.0202	0.087*	
H13C	1.0433	0.7813	0.0863	0.087*	
H1A	0.734 (2)	0.234 (2)	−0.030 (3)	0.048 (7)*	

### Atomic displacement parameters (Å^2^)

**Table:**

	*U*^11^	*U*^22^	*U*^33^	*U*^12^	*U*^13^	*U*^23^
S1	0.0650 (4)	0.0340 (3)	0.0487 (4)	0.0064 (3)	0.0070 (3)	−0.0051 (2)
O1	0.0643 (10)	0.0483 (9)	0.0242 (7)	−0.0124 (8)	0.0019 (6)	0.0034 (6)
O2	0.0803 (12)	0.0404 (9)	0.0530 (10)	−0.0216 (9)	−0.0036 (9)	0.0101 (7)
N1	0.0480 (10)	0.0322 (9)	0.0237 (8)	−0.0064 (7)	0.0070 (7)	−0.0009 (7)
N2	0.0454 (10)	0.0273 (8)	0.0427 (10)	−0.0038 (7)	0.0077 (8)	0.0025 (7)
C1	0.0527 (13)	0.0360 (11)	0.0267 (10)	−0.0074 (9)	−0.0005 (9)	−0.0014 (8)
C2	0.0712 (16)	0.0421 (12)	0.0279 (10)	−0.0107 (11)	−0.0019 (10)	0.0062 (9)
C3	0.0428 (11)	0.0319 (10)	0.0415 (12)	−0.0035 (9)	0.0028 (9)	0.0038 (9)
C4	0.0489 (12)	0.0407 (12)	0.0360 (11)	−0.0089 (10)	−0.0033 (9)	−0.0059 (9)
C5	0.0473 (12)	0.0423 (12)	0.0250 (9)	−0.0068 (9)	−0.0010 (8)	−0.0006 (8)
C6	0.0293 (9)	0.0306 (9)	0.0271 (9)	0.0000 (7)	0.0030 (7)	0.0001 (7)
C7	0.0350 (10)	0.0332 (10)	0.0251 (9)	0.0007 (8)	0.0039 (7)	0.0004 (7)
C8	0.0405 (11)	0.0317 (10)	0.0236 (9)	−0.0024 (8)	−0.0011 (8)	−0.0005 (7)
C9	0.0539 (14)	0.0379 (12)	0.0614 (15)	0.0057 (10)	0.0207 (11)	0.0027 (11)
C10	0.0517 (15)	0.0604 (17)	0.101 (2)	0.0007 (13)	0.0229 (15)	−0.0096 (16)
C11	0.0720 (19)	0.0608 (17)	0.095 (2)	−0.0163 (15)	0.0387 (17)	0.0083 (16)
C12	0.0486 (13)	0.0379 (11)	0.0543 (14)	−0.0136 (10)	0.0037 (11)	0.0074 (10)
C13	0.0617 (16)	0.0372 (13)	0.0752 (18)	−0.0113 (11)	−0.0010 (13)	−0.0021 (12)

### Geometric parameters (Å, º)

**Table:**

S1—C8	1.662 (2)	C5—C6	1.386 (3)
O1—C7	1.220 (2)	C5—H5	0.9300
O2—C3	1.361 (2)	C6—C7	1.482 (3)
O2—C13	1.418 (3)	C9—C10	1.505 (4)
N1—C7	1.372 (2)	C9—H9A	0.9700
N1—C8	1.415 (2)	C9—H9B	0.9700
N1—H1A	0.83 (3)	C10—C11	1.512 (4)
N2—C8	1.319 (3)	C10—H10A	0.9700
N2—C12	1.471 (3)	C10—H10B	0.9700
N2—C9	1.472 (3)	C11—C12	1.502 (4)
C1—C2	1.375 (3)	C11—H11A	0.9700
C1—C6	1.387 (3)	C11—H11B	0.9700
C1—H1	0.9300	C12—H12A	0.9700
C2—C3	1.384 (3)	C12—H12B	0.9700
C2—H2	0.9300	C13—H13A	0.9600
C3—C4	1.380 (3)	C13—H13B	0.9600
C4—C5	1.381 (3)	C13—H13C	0.9600
C4—H4	0.9300		
			
C3—O2—C13	118.55 (19)	N2—C9—C10	103.63 (19)
C7—N1—C8	121.83 (16)	N2—C9—H9A	111.0
C7—N1—H1A	119.3 (18)	C10—C9—H9A	111.0
C8—N1—H1A	114.0 (18)	N2—C9—H9B	111.0
C8—N2—C12	122.12 (18)	C10—C9—H9B	111.0
C8—N2—C9	126.67 (17)	H9A—C9—H9B	109.0
C12—N2—C9	111.08 (17)	C9—C10—C11	103.0 (2)
C2—C1—C6	120.23 (19)	C9—C10—H10A	111.2
C2—C1—H1	119.9	C11—C10—H10A	111.2
C6—C1—H1	119.9	C9—C10—H10B	111.2
C1—C2—C3	120.82 (19)	C11—C10—H10B	111.2
C1—C2—H2	119.6	H10A—C10—H10B	109.1
C3—C2—H2	119.6	C12—C11—C10	104.4 (2)
O2—C3—C4	124.7 (2)	C12—C11—H11A	110.9
O2—C3—C2	115.90 (19)	C10—C11—H11A	110.9
C4—C3—C2	119.41 (19)	C12—C11—H11B	110.9
C3—C4—C5	119.72 (19)	C10—C11—H11B	110.9
C3—C4—H4	120.1	H11A—C11—H11B	108.9
C5—C4—H4	120.1	N2—C12—C11	103.30 (19)
C4—C5—C6	121.15 (19)	N2—C12—H12A	111.1
C4—C5—H5	119.4	C11—C12—H12A	111.1
C6—C5—H5	119.4	N2—C12—H12B	111.1
C5—C6—C1	118.65 (18)	C11—C12—H12B	111.1
C5—C6—C7	117.66 (17)	H12A—C12—H12B	109.1
C1—C6—C7	123.61 (17)	O2—C13—H13A	109.5
O1—C7—N1	120.88 (18)	O2—C13—H13B	109.5
O1—C7—C6	121.76 (18)	H13A—C13—H13B	109.5
N1—C7—C6	117.32 (16)	O2—C13—H13C	109.5
N2—C8—N1	115.53 (17)	H13A—C13—H13C	109.5
N2—C8—S1	124.18 (15)	H13B—C13—H13C	109.5
N1—C8—S1	120.28 (15)		
			
C6—C1—C2—C3	0.2 (4)	C5—C6—C7—N1	179.28 (18)
C13—O2—C3—C4	1.7 (4)	C1—C6—C7—N1	2.5 (3)
C13—O2—C3—C2	−178.4 (2)	C12—N2—C8—N1	−176.49 (18)
C1—C2—C3—O2	178.9 (2)	C9—N2—C8—N1	8.0 (3)
C1—C2—C3—C4	−1.2 (4)	C12—N2—C8—S1	4.8 (3)
O2—C3—C4—C5	−178.5 (2)	C9—N2—C8—S1	−170.72 (18)
C2—C3—C4—C5	1.5 (3)	C7—N1—C8—N2	63.0 (3)
C3—C4—C5—C6	−0.9 (3)	C7—N1—C8—S1	−118.27 (18)
C4—C5—C6—C1	−0.1 (3)	C8—N2—C9—C10	162.6 (2)
C4—C5—C6—C7	−177.08 (19)	C12—N2—C9—C10	−13.4 (3)
C2—C1—C6—C5	0.5 (3)	N2—C9—C10—C11	31.6 (3)
C2—C1—C6—C7	177.3 (2)	C9—C10—C11—C12	−38.8 (3)
C8—N1—C7—O1	−2.6 (3)	C8—N2—C12—C11	173.3 (2)
C8—N1—C7—C6	179.56 (17)	C9—N2—C12—C11	−10.5 (3)
C5—C6—C7—O1	1.5 (3)	C10—C11—C12—N2	30.2 (3)
C1—C6—C7—O1	−175.3 (2)		

### Hydrogen-bond geometry (Å, º)

**Table:**

*D*—H···*A*	*D*—H	H···*A*	*D*···*A*	*D*—H···*A*
N1—H1*A*···O1^i^	0.83 (3)	2.11 (3)	2.927 (2)	170 (2)
C1—H1···O1^i^	0.93	2.50	3.350 (3)	152

Symmetry code: (i) *x*, −*y*+1/2, *z*−1/2.
