# Supplementary figures and images for: Crystal structure of 4-meth­oxy-N-[(pyrrolidin-1-yl)carbo­thio­yl]benzamide
Source: Acta Crystallogr E Crystallogr Commun. 2015 Mar 4;71(Pt 4):o225–6. doi: 10.1107/S2056989015003813 (PMC4438823; doi:10.1107/S2056989015003813)

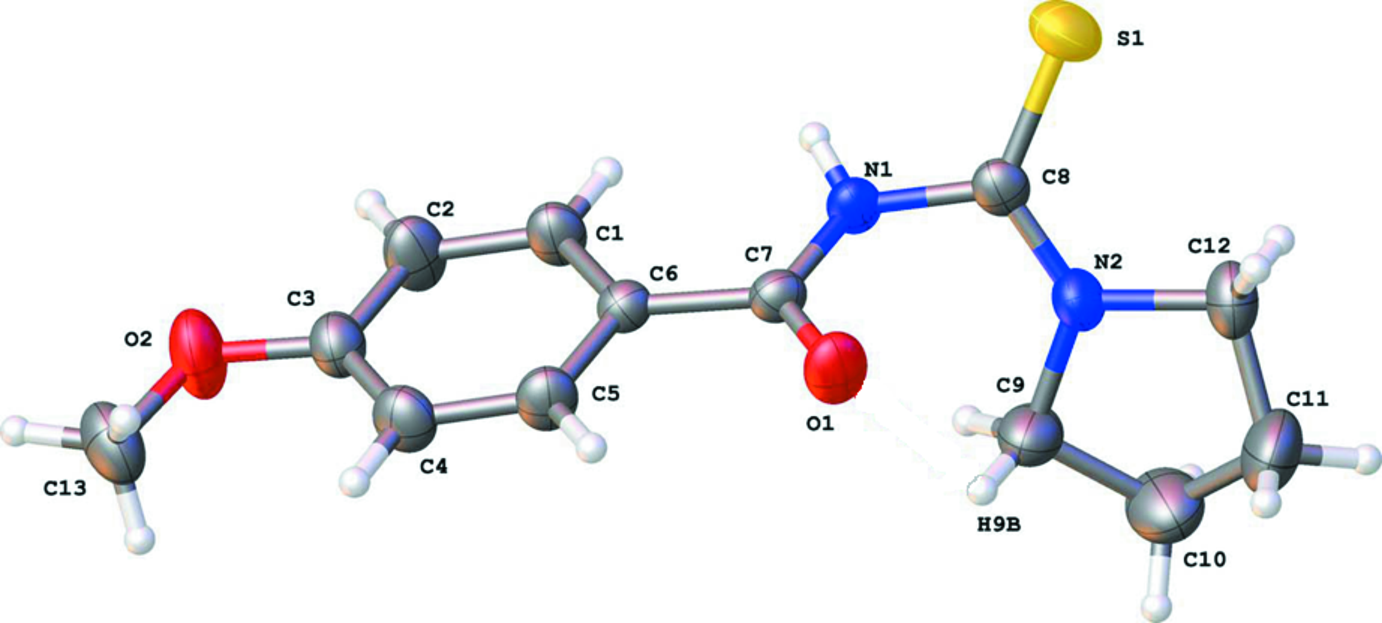

Supplement: Supplementary file 4 [file e-71-0o225-fig1.tif]

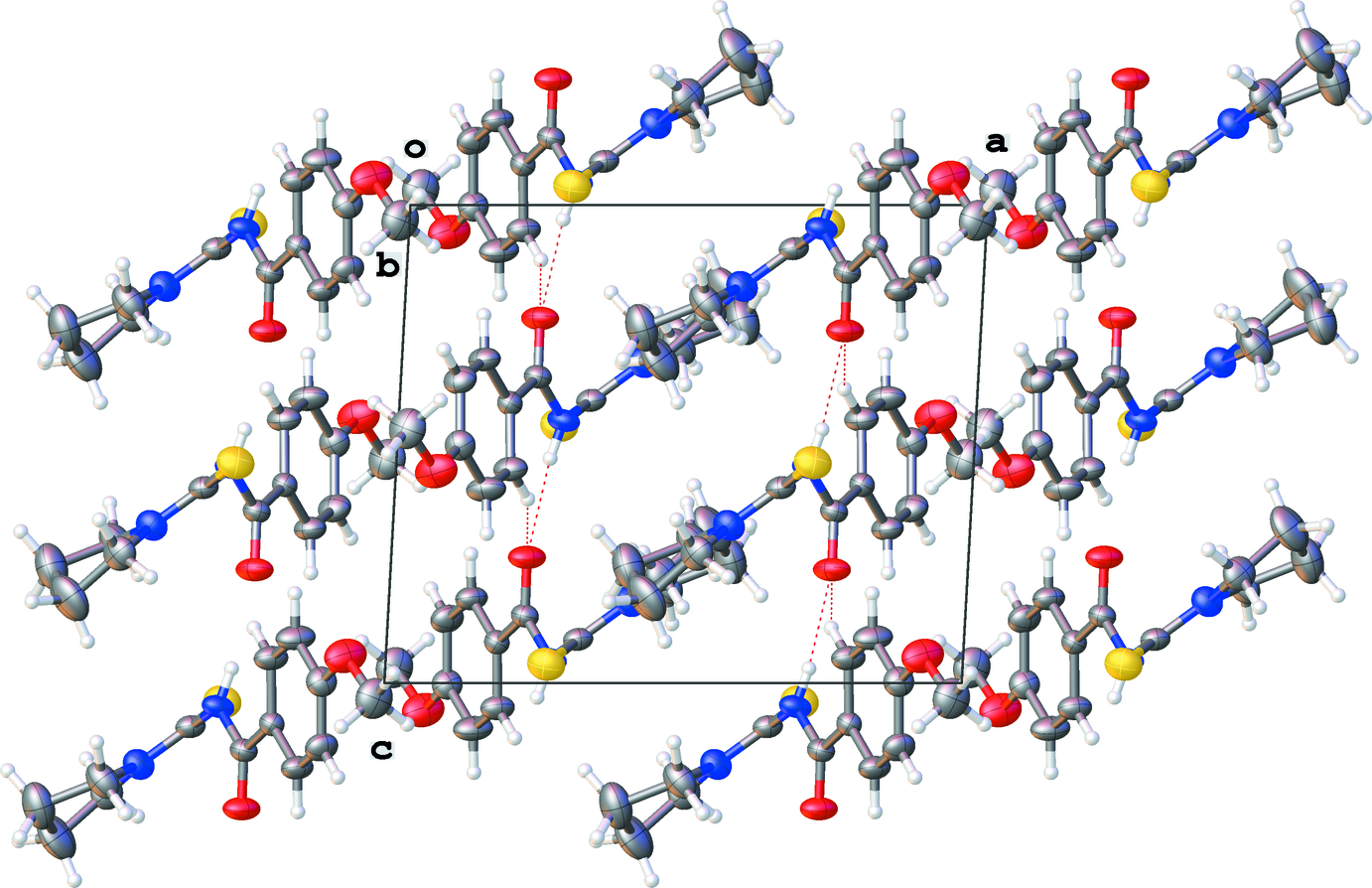

Supplement: Supplementary file 5 [file e-71-0o225-fig2.tif]
